# Multiple Logistic Regression Analysis of Smartphone Use in University Students

**DOI:** 10.3389/fpsyg.2022.821345

**Published:** 2022-03-29

**Authors:** Chen-Shen Chao, Ming-Hsien Li, Shih-Pei Chang, Yu-Hsuan Chen

**Affiliations:** ^1^Office of Physical Education and Sport Affairs, Feng Chia University, Taichung, Taiwan; ^2^Department of Medical Laboratory Science and Biotechnology, Central Taiwan University of Science and Technology, Taichung, Taiwan; ^3^Department of Physical Education, Central Taiwan University of Science and Technology, Taichung, Taiwan

**Keywords:** smartphone, exercise participation, smartphone addiction, perceived exercise efficacy, Pittsburgh Sleep Quality Index (PSQI)

## Abstract

Problematic smartphone use (PSU) is an expanded public health heed that requires more study to clarify the influence elements of different populations. The aim of this study was to investigate the relationship between smartphone use, and sleep quality, self-perceived health, and exercise participation in university students. A total of 1,575 Taiwanese undergraduate students from 7 universities participated in the study. Three questionnaires were completed by the study individuals. The results show the overall PSU rate was 11.8%. Average smartphone users were more likely to feel in good health, better sleep quality and less unsatisfactory exercise participation than those who were problematic smartphone users. Multiple logistic regression analysis indicated that PSU, low weekly exercise frequency, and poor sleep quality were significant indicators of poor self-perceived health. We concluded that both low physical activity and PSU did have negative impacts on self-perceived health and sleep quality for undergraduate students.

## Introduction

Smartphones play a very important role in modern life. They are not only a necessity in modern social life but also an important tool for the workplace and communication. Social networking applications are now common communication tools among schools, teachers, students, parents, friends, and work associates. With the improvement of smartphone functions, the motivation of smartphone use has changed from purely interpersonal communication to a tool that can make people feel safe, contact others at any time, and provide entertainment functions (Wei, 2008). A smartphone is like a personal computer that can be taken away. It has become an indispensable tool in people’s lives and workplaces. Smartphones can expand the opportunities for interpersonal communication and social networking (Igarashi et al., 2005). In 2011, smartphones became widely available, since then usage has accumulated. According to the report from Pew Research Center demonstrated that more than 2.5 billion people have smartphones (Yang et al., 2020). Smartphone is becoming a daily necessity for people because its convenience to gain information, social interaction, workplace apps and entertainment (Yang et al., 2020). However, this popularity has translated to the overuse of smartphones, which has become a public health problem worldwide. Problematic smartphone use (PSU) was reported in approximately one in every four children and young people with poorer mental health (Sohn et al., 2019). Positive correlations have been revealed between smartphone overuse and depression, anxiety, poor sleep quality (Demirci et al., 2015; Elhai et al., 2017; Matar and Jaalouk, 2017). In addition to, physical activity, poor academic achievement, number of hours per day spent using smartphones, years of undergraduate study (Haug et al., 2015; Kim and Kim, 2015; Alosaimi et al., 2016) and problematic decision-making (Tang et al., 2017) also affected. Many medical problems such as muscular pain and disturbance, damaged hand functions, and injuries of the neck and spine caused by smartphone overuse (İnal et al., 2015; Kim and Kim, 2015; Kee et al., 2016; Megna et al., 2017). Several studies have reported a significant gender effect of smartphone use. Females are more prone to smartphone addiction than males (Demirci et al., 2015; Kim et al., 2015; Lee et al., 2016; Randler et al., 2016).

Except gender, poor self-perceiving health, sleep onset after 12 am, longer sleep latency and sleep time less than 6 h are also the risk factors of smartphone addiction in Korean adolescents (Chung et al., 2018). In a cross-sectional survey, six hundred and eighty-six middle and high school students, indicate sleep quality mediated the relationship between PSU and health symptoms (Xie et al., 2018). In UK cross-sectional study of young adults, those who with smartphone addiction, 68.7% had poor sleep quality, more than those without (57.01%) (Sohn et al., 2021). It is quite common for college students to use smartphones. The degree of smartphone use does affect their sleep quality. Excessive use of smartphones can also cause physical and psychological problems. In addition to affecting the vitality of individuals, it may also affect the mental health of students (Beranuy et al., 2009), and female students are more likely to be at risk of smartphone addiction than male students (Billieux et al., 2007), and may cause problems such as anxiety and insomnia (Jenaro et al., 2007). Yang reported a systematic review and meta-analysis of PSU related articles, they found significantly increased risks of poor sleep quality, depression, and anxiety in people with PSU. They suggest highlight of managing the PSU is necessary (Yang et al., 2020). Regular exercise is known to be beneficial for physical and mental health. Regular exercise can improve quality of life, sleep, mood, and health conditions of elderly and patients with diseases, including cancer, depression, anxiety, insomnia, and other disorders (Reid et al., 2010; Halpern et al., 2014). PSU have occupied time from other health-related activities such as physical activity and social interactions (Thomée, 2018). Exercise has also been proposed as a form of rehabilitation for smartphone addiction (Kim, 2013). Previous studies show that physical exercise may reduce smartphone addiction (Liu et al., 2019). The other study indicate that inactive physical adolescent students were more problematic smartphone users than those who were active physical students (Pereira et al., 2020). A previous survey show reveals the frequency of exercise was significantly correlated with sleep quality and self-perceived health in Taiwanese university students (Chang et al., 2016). However, the problematic smartphone uses, exercise participation and quality of life are not yet clear in Taiwan.

After the popularity of smartphones, excessive use of smartphones often leads to sleep disorders or insomnia, and even headaches, shoulder and neck pain. Taiwan has a high rate of smartphone use (65.4%), but studies of smartphone overuse and its impact on university students are scarce. How high rate of smartphone use affects exercise participation, sleep quality, and self-perceived health of university students remains unclear. Therefore, this study aimed to find out the prevalence of problematic smartphone usage and the relationships between exercise participation behavior, sleep quality, and self-perceived health among university students.

## Materials and Methods

### Participants

A cross-sectional survey was conducted between March and May 2017 in 7 universities in Taiwan. A cluster sampling was adopted and all students who attended the physical education (PE) classes who participated in the current study. Of the 1,700 questionnaires distributed, 1,575 were completed by the students (17–27 years of age), which questionnaire response rate of 92.6%. All study participants provided signed written informed consent before completing the study questionnaire and assessment scales. This study was approved by the University Medical Research Ethics Committee of the researchers’ institution (No. 10512004).

### Demographic Characteristics

A self-administered structured questionnaire was used to collect information on age, gender, and self-perceived health. Height and body weight were measured by the PE teachers during classes. Students with a body mass index (BMI) equal to or above 24 kg/m^2^ were considered overweight on the basis of the guidelines of the Health Promotion Administration of Taiwan’s Ministry of Health and Welfare (2016).

### Exercise Participation

Exercise participation behaviors, including the frequency, duration, intensity, and satisfaction were assessed using the Exercise Participation of University Students questionnaire proposed by the Ministry of Education within the past 3 months (Ministry of Education, 2010). Exercise frequency and cumulative exercise hours per week were categorized into 3 groups (i.e., ≤1, 2–3, and > 3 d/week; and ≤1, 1–2.5, > 2.5 h/week, respectively), including frequency (3 days a week), duration (more than 30 min each time), intensity (130 beats per minute).

### Smartphone Addiction

The level of smartphone addiction was evaluated using a Chinese version of a 20-item questionnaire revised from the Internet Addiction Test by Dr. Kimberly Young (1998). A Likert-type scale was used for scoring: scores of 1 and 5 reflect the lowest and highest frequencies, respectively. Participants with global smartphone addiction scores (SAS) that ranged from 20 to 49 were considered average users; scores of 50–79 denoted occasional or frequent problematic users, and scores of 80 or higher denoted significant problematic users. The number of participants in the significant problematic user group was small, so participants with global scores of 50 or higher were combined into a single group (i.e., problematic users). The Cronbach’s alpha coefficient for the global score in this study was 0.891, indicating high internal consistency.

### Sleep Quality

The sleep quality of participants was assessed using the Pittsburgh Sleep Quality Index (PSQI). Seven dimensions were measured: subjective sleep quality, sleep latency, sleep duration, habitual sleep efficiency, sleep disturbances, use of sleep medication, and daytime dysfunction over the past month. The scoring system used a Likert-type scale with a score of 3 reflecting a negative extreme. Participants who had a global score of greater than 5 were defined as poor-quality sleepers; those with a score of 5 or below were defined as good-quality sleepers. The original scale yielded diagnostic sensitivity and specificity of 89.6 and 86.5%, respectively, and the Cronbach’s alpha coefficient was 0.83, which indicated high internal consistency (Buysse et al., 1989).

### Statistical Analysis

Independent 2-sample *t*-tests were used for continuous variable comparisons between genders and the 2 categories of smartphone use. *Chi-square* tests were performed for categorical variable comparisons. The linear associations among BMI, weekly exercise frequency, SAS, and PSQI score were analyzed using Spearman correlation coefficients. Thereafter, multiple logistic regression analysis with forward model selection was conducted to determine whether gender, BMI, exercise participation, and problematic smartphone use (PSU) were independent indicators for self-perceived health and sleep quality. All statistical analyses were performed using SPSS 18.0 for Windows (IBM, Somers, NY, United States) and the level of significance was set at 0.05.

## Results

A total of 764 males and 811 females with a mean age of 19.5 years (*SD* = 1.3) were enrolled in this study. BMI, self-perceived health status, exercise participation, SAS, and sleep quality were compared between genders (Table 1). The male students had significantly higher BMI and higher percentages of overweight (BMI ≥ 24 kg/m^2^). Self-perceived health, frequency of exercise, and satisfactory exercise participation male students also better than female students (*p* < 0.001). Although the original SAS was not significantly different between genders, male students had a significantly higher percentage of problematic users than female students (13.7% for males vs. 10.0% for females, *p* = 0.021). The males also reported have better sleep quality (global PSQI score 5.35 ± 2.72 for males vs. 5.63 ± 2.88 for females, *p* = 0.042). However, the percentages of males and females with poor sleep quality were not significantly different (42.5% for males vs. 47% for females, *p* = 0.077).

**TABLE 1:** Gender comparison of BMI, self-perceived health status, exercise participation, smartphone addiction score, and sleep quality.

	Male (*n* = 764)		Female (*n* = 811)		
	N	%	N	%	*p*
**BMI (kg/m^2^)**					
Mean (SD)	22.3	(4.0)	20.9	(3.5)	<0.001
<18.5	105	13.7	196	24.2	<0.001
18.5–23.9	445	58.2	499	61.5	
≥24	214	28.0	116	14.3	
Self-perceived health					<0.001
Good	485	63.5	395	48.7	
Average	250	32.7	383	47.2	
Poor	29	3.8	33	4.1	
Join sport club	323	42.3	199	24.5	<0.001
**Exercise frequency (d/wk)**					
Mean (SD)	2.8	(1.7)	2.2	(1.5)	<0.001
≤1	179	23.4	319	39.3	<0.001
2–3	381	49.9	361	44.5	
>3	204	26.7	131	16.2	
Weekly exercise time					<0.001
≤1 h	118	15.4	234	28.9	
1–2.5 h	270	35.3	374	46.1	
>2.5 h	376	49.2	203	25.0	
Exercise intensity					<0.001
Mild	112	14.7	157	19.4	
Moderate	452	59.2	539	66.5	
Vigorous	200	26.2	115	14.2	
Satisfaction with exercise participation				<0.001	
Unsatisfied	96	12.6	158	19.5	
Moderately satisfied	401	52.5	502	61.9	
Satisfied	267	34.9	151	18.6	
**Smartphone addiction score**					
Mean (SD)	37.4	(11.3)	37.0	(10.2)	0.438
Average users (< 50)	659	86.3	730	90.0	0.021
Problematic users (≥ 50)	105	13.7	81	10.0	
**Sleep quality**					
Global PSQI Mean (SD)	5.35	(2.72)	5.63	(2.88)	0.042
Good (PSQI ≤ 5)	439	57.5	430	53.0	0.077
Poor (PSQI > 5)	325	42.5	381	47.0	

*BMI, body mass index; PSQI, Pittsburgh Sleep Quality Index. P-value by 2-independent t-test or chi-square test when appropriate.*

Average smartphone users had a higher percentage of Self-perceived health and a lower percentage of dissatisfaction with exercise participation (*p* < 0.001) than problematic users. Although no significant differences were observed in terms of joining sport clubs, exercise frequency, and weekly exercise time, problematic smartphone users had a higher proportion of participants who engaged in vigorous exercise than average users (30.1% vs. 18.6%, *p* < 0.001). In addition, problematic users had a significantly higher percentage of poor-quality sleepers (65.1% vs. 42.1%, *p* < 0.001) and higher global PSQI score (6.76 ± 3.20 vs. 5.32 ± 2.71, *p* < 0.001) than average users (Table 2).

**TABLE 2:** Comparison of BMI, self-perceived health status, exercise participation and sleep quality between average and problematic smartphone users.

	Average users (*n* = 1389)		Problematic users (*n* = 186)		
	N	%	N	%	*p*
**BMI (kg/m^2^)**					
Mean (SD)	21.6	(3.8)	21.6	(4.1)	0.907
<18.5	263	18.9	38	20.4	0.886
18.5–23.9	834	60.0	110	59.1	
≥ 24	292	21.0	38	20.4	
Self-perceived health					<0.001
Good	781	56.2	99	53.2	
Average	563	40.5	70	37.6	
Poor	45	3.2	17	9.1	
Join sport club	461	33.2	61	32.8	0.915
**Exercise frequency (d/wk)**					
Mean (SD)	2.5	(1.6)	2.4	(1.8)	0.593
≤1	435	31.3	63	33.9	0.626
2–3	654	47.1	88	47.3	
>3	300	21.6	35	18.8	
Weekly exercise time					0.533
≤1 h	308	22.2	44	23.7	
1–2.5 h	575	41.4	69	37.1	
>2.5 h	506	36.4	73	39.2	
Exercise intensity					<0.001
Mild	236	17.0	33	17.7	
Moderate	894	64.4	97	52.2	
Vigorous	259	18.6	56	30.1	
Satisfaction with exercise participation				0.002	
Unsatisfied	208	15.0	46	24.7	
Moderately satisfied	812	58.5	91	48.9	
Satisfied	369	26.6	49	26.3	
**Sleep quality**					
Global PSQI Mean (SD)	5.32	(2.71)	6.76	(3.20)	<0.001
Good (PSQI ≤ 5)	804	57.9	65	34.9	<0.001
Poor (PSQI > 5)	585	42.1	121	65.1	

*BMI, body mass index; PSQI, Pittsburgh Sleep Quality Index.*

*P-value by 2-independent t-test or chi-square test when appropriate.*

The linear associations assessed by Pearson correlation coefficients among BMI, weekly exercise frequency, SAS, and PSQI score are summarized in Table 3. SAS was positively correlated with PSQI score for both genders (*r* = 0.239, *p* < 0.01), which indicates that the more students were addicted to smartphones, the worse their sleep quality became. Weekly exercise frequency was positively associated with BMI for females and all participants (*p* < 0.01). Although the correlation coefficients stratified by gender were not significant, weekly exercise frequency was negatively associated with global PSQI score for all participants (*r* = –0.056, *p* = 0.025). This result indicates that the students who exercised less frequently were more likely to have worse sleep quality.

**TABLE 3:** Pearson correlation coefficient among BMI, weekly exercise frequency, smartphone addiction score and global PSQI score.

Pearson correlation coefficient	Weekly exercise frequency	Smartphone addiction score	Global PSQI score
**Male (*N* = 764)**			
BMI	0.053	−0.015	−0.036
Weekly exercise frequency		−0.068	−0.059
Smartphone addiction score			0.239**
**Female (*N* = 811)**			
BMI	0.096**	0.019	0.031
Weekly exercise frequency		−0.016	−0.036
Smartphone addiction score			0.239**
**All (*N* = 1,575)**			
BMI	0.105**	0.004	−0.012
Weekly exercise frequency		−0.040	−0.056*
Smartphone addiction score			0.237**

BMI, body mass index, PSQI, Pittsburgh Sleep Quality Index. *p < 0.05; **p < 0.01.

Table 4 presents the results of the multiple logistic regression analysis for the indicators of self-perceived health and sleep quality. The results showed that individuals who exercised 2–3 days each week were less likely to feel unhealthy than those who exercised 1 or fewer days each week [odds ratio (OR) = 0.45, *p* = 0.012]. However, those who exercised more than 3 days each week did not show significantly better self-perceived health than those whose weekly exercise frequency was 1 or fewer days. PSU and poor sleep quality were significant indicators of poor health (OR = 2.46, *p* = 0.004; OR = 3.41, *p* < 0.001, respectively). PSU (OR = 2.42, *p* < 0.001) and poor health (OR = 3.22, *p* < 0.001) were also significantly associated with poor sleep quality. Students who were more satisfied with their exercise participation were less likely to have poor sleep quality than those who felt dissatisfied with exercise participation (OR = 0.65, *p* = 0.008). Gender, BMI, weekly exercise time, and exercise intensity were not significant independent indicators of self-perceived health and sleep quality after the model selection.

**TABLE 4:** Result of multiple logistic regression with forward model selection.

	Odds ratio	95% *CI*	*P*
**Dependent variable: Self-perceived health (Poor vs. Good/Average)*^a^***			
BMI 18.5–23.9 vs. < 18.5	0.52	(0.26–1.03)	0.061
BMI ≥ 24 vs. < 18.5	1.76	(0.88–3.53)	0.112
Weekly exercise frequency 2–3 vs. ≤ 1day/week	0.45	(0.25–0.84)	0.012
Weekly exercise frequency > 3 vs. 1 day/week	0.93	(0.48–1.79)	0.823
Problematic smartphone users vs. average users	2.46	(1.34–4.49)	0.004
PSQI (>5 Poor vs. ≤ 5 Good sleep quality)	3.41	(1.89–6.16)	<0.001
**Dependent variable: PSQI score (> 5 Poor vs. 5 Good sleep quality)*^b^***			
Moderately satisfied vs. unsatisfied with exercise participation	0.89	(0.67–1.18)	0.416
Satisfied vs. unsatisfied with exercise participation	0.65	(0.47–0.89)	0.008
Problematic smartphone users vs. average users	2.42	(1.75–3.35)	<0.001
Self-perceived health (Poor vs. Good/Average)	3.22	(1.79–5.79)	<0.001

*BMI, body mass index; PSQI, Pittsburgh Sleep Quality Index; CI, confidence interval.*

*^a^Independent variables included gender, BMI, weekly exercise frequency, exercise intensity, weekly exercise time, satisfaction with exercise participation, smartphone use group, and sleep quality.*

*^b^Independent variables included gender, BMI, weekly exercise frequency, exercise intensity, weekly exercise time, satisfaction with exercise participation, and smartphone use group.*

## Discussion

Although Taiwan has a high rate of smartphone use and widespread access to wireless internet services, the overall PSU rate (11.8%) in this study was lower than that of adolescents and university students in other countries (16.9–71.9%) (Davey and Davey, 2014; Haug et al., 2015; Alosaimi et al., 2016; Long et al., 2016; Venkatesh et al., 2017). The discrepancy may cause by inter-country variation or inconsistent assessment tools used. The variation between adolescents and university students who are substantially addicted to smartphones may account for such discrepancies (Haug et al., 2015; Randler et al., 2016). A Korean study that compared the risk factors for smartphone and internet addiction showed that increased SAS were associated with females, but increased internet addiction scores were associated with males (Choi et al., 2015). This phenomenon is related to the purposes of smartphone use: females mainly used smartphones for social networking and males tended to use computers for online gaming, which could not be easily replaced by smartphones (Choi et al., 2015; Demirci et al., 2015). However, contrary to the conclusions from other studies, the current study observed a significantly higher rate of PSU in male students than in female students (13.7% for males vs. 10.0% for females, *p* = 0.021). One possible explanation is that the limitations for using smartphones for online gaming have been gradually overcome due to improvements in online gaming applications developed for smartphones. The percentages of students who did not exercise regularly were slightly lower (23.4% for males and 39.3% for females) than those of a previous study (29.0% for males and 44.2% for females) (Chang et al., 2016). However, significant gender variations were also observed in self-perceived health, exercise participation, and sleep quality. Males have higher scores than females in most of these categories. Although male students had significantly higher weekly exercise frequency than females, a significant positive correlation was observed between BMI and exercise frequency in females but not in males. A possible explanation is that females were more likely to be motivated to exercise by body image than males, i.e., the female students who had higher BMI tended to exercised more.

Not surprisingly, problematic smartphone users had a significantly higher percentage of poor self-perceived health and feeling unsatisfied with exercise participation than average smartphone users. This indicates the negative effects of smartphone use on health and exercise participation. Some researchers have reported that high-risk smartphone users engaged in limited physical activity (Haug et al., 2015; Kim et al., 2015; Alosaimi et al., 2016). But, in this study no significant difference was observed between average and problematic smartphone users in frequency or cumulative time of exercise. Conversely, exercise intensity was determined to be significantly correlated with smartphone use. Problematic users tended to perform more vigorous exercise than average users (*p* < 0.001). This result showed that the use of smartphones may directly affect not the amount of time but the intensity of exercise for Taiwanese university students. This finding implies that students who were considerably addicted to smartphones were prone to enjoy such activities as vigorous exercise. We also found that no significant correlation was observed between SAS and both BMI and exercise frequency. Our result was same as Prabhu and Abishek (2019) they found that there is no significant association between smartphone addiction and BMI amongst dental students. But Deniz (2019) found there is a relationship between smartphone use and overweight. Smart phone addiction causes overweight twice as much as the average person.

High mobile phone use has been reported to be associated with a later bedtime (Lemola et al., 2015). Demirci et al. (2015) indicated that sleep quality, sleep disturbance, and daytime dysfunction were positively correlated with SAS. Lee et al. (2017) reported that high mobile phone addiction increased the risk of poor sleep quality but not short sleep duration. In the present study, a significant correlation was confirmed between smartphone use and sleep quality. Both male and female students with high SAS tended to have high global PSQI scores (*p* < 0.001). Problematic smartphone users had significantly worse sleep quality than average smartphone users. Students with limited weekly exercise frequency seemed to have worse sleep quality (i.e., high PSQI score, *r* = −0.056, *p* < 0.05). There was no significant association between frequency and amount of exercise and SAS. In general, the sleep quality was affected directly by smartphone use rather than through exercise participation among participants.

The multiple logistic regression results indicated that low weekly exercise frequency (≤1 day/week), PSU, and poor sleep quality were significant indicators of poor self-perceived health. On the other hand, dissatisfaction with exercise participation, PSU, and poor health were significantly associated with poor sleep quality. Both models confirmed that exercise participation and smartphone use are important factors for both health and sleep quality among university students. Self-perceived health and sleep quality are both subjective evaluations and may be affected by many factors, including physical, mental, and environmental factors, but exercise and smartphone use did affect individuals’ self-perceptions. Regular exercise at the suggested frequency of 2–3 days per week and moderate smartphone use would be beneficial for university students in terms of health and sleep quality. Moreover, exercising more than 3 days each week showed no additional advantage for health compared to exercising 1 or fewer days each week. This implies that adequate exercise, but not over-exercise, would improve the health of students. In terms of sleep quality, satisfaction with exercise participation seemed more important than the amount and intensity of exercise. This result suggests that the mental reward from exercise is substantially beneficial to sleep quality.

A few limitations in this study must be noted. First, the health status of the study participants was assessed by personal perception without physical examinations or uniform standards. Second, information on exercise participation was gathered *via* a 1-time self-administered questionnaire and measurement error may have occurred in our exercise assessment. For example, exercise behavior may vary before or after examinations or at the beginning or end of semesters.

This study demonstrated an overall PSU rate of 11.8% among university students, with males having a higher rate than females. The use of smartphones was significantly associated with the intensity of exercise, and a significant positive correlation was observed between BMI and weekly exercise frequency. Both BMI and weekly exercise frequency were not significantly associated with SAS. Low weekly exercise frequency (≤1 day/week), PSU, and poor sleep quality were significant indicators of poor self-perceived health. Dissatisfaction with exercise participation, PSU, and poor health were significantly associated with poor sleep quality. In summary, both low physical activity and PSU did have negative impacts on self-perceived health and sleep quality for undergraduate students. Paying more attention to the issues of smartphone use and insufficient exercise would assist young adults to improve their health and sleep quality.

## Data Availability Statement

The original contributions presented in the study are included in the article/supplementary material, further inquiries can be directed to the corresponding author/s.

## Ethics Statement

The studies involving human participants were reviewed and approved by the Asia University Medical Research Ethics Committee. Written informed consent for participation was not required for this study in accordance with the national legislation and the institutional requirements.

## Author Contributions

C-SC and Y-HC contributed rate of 100%. M-HL contributed rate of 80%. S-PC contributed of rate of 60%. All authors contributed to the article and approved the submitted version.

## Conflict of Interest

The authors declare that the research was conducted in the absence of any commercial or financial relationships that could be construed as a potential conflict of interest.

## Publisher’s Note

All claims expressed in this article are solely those of the authors and do not necessarily represent those of their affiliated organizations, or those of the publisher, the editors and the reviewers. Any product that may be evaluated in this article, or claim that may be made by its manufacturer, is not guaranteed or endorsed by the publisher.
